# Effects of question formats on causal judgments and model evaluation

**DOI:** 10.3389/fpsyg.2015.00467

**Published:** 2015-04-21

**Authors:** Yiyun Shou, Michael Smithson

**Affiliations:** Research School of Psychology, The Australian National UniversityCanberra, ACT, Australia

**Keywords:** causal reasoning, judgment, measurement, question formats, causal models

## Abstract

Evaluation of causal reasoning models depends on how well the subjects’ causal beliefs are assessed. Elicitation of causal beliefs is determined by the experimental questions put to subjects. We examined the impact of question formats commonly used in causal reasoning research on participant’s responses. The results of our experiment (Study 1) demonstrate that both the mean and homogeneity of the responses can be substantially influenced by the type of question (structure induction versus strength estimation versus prediction). Study 2A demonstrates that subjects’ responses to a question requiring them to predict the effect of a candidate cause can be significantly lower and more heterogeneous than their responses to a question asking them to diagnose a cause when given an effect. Study 2B suggests that diagnostic reasoning can strongly benefit from cues relating to temporal precedence of the cause in the question. Finally, we evaluated 16 variations of recent computational models and found the model fitting was substantially influenced by the type of questions. Our results show that future research in causal reasoning should place a high priority on disentangling the effects of question formats from the effects of experimental manipulations, because that will enable comparisons between models of causal reasoning uncontaminated by method artifact.

## Introduction

Historically, researchers in cognitive science have been interested in developing psychological models of human causal reasoning. A number of quantitative models have been proposed to explain how people reason causal relationships from covariance information (Hattori and Oaksford, 2007). Several recent studies have compared the accuracies of different models in predicting human causal judgments (e.g., Cheng, 1997; Hattori and Oaksford, 2007; Perales and Shanks, 2007; Lu et al., 2008; Carroll et al., 2013). Results of these comparisons varied across different studies using data from different experiments, with various question formats. The inconsistent results in the model comparison studies may be contributed to by the variation in the responses across different experiments. One possible factor is the variation in the experimental procedures, as people’s responses can be influenced by the methods of presenting stimuli (Vallée-Tourangeau et al., 2008) and the experimental instructions (Matute, 1996; White, 2003). For example, subjects in experiments that presented the stimuli trial-by-trial (e.g., Hattori and Oaksford, 2007; Perales and Shanks, 2007) were less likely to retain all observed evidence than subjects in the experiments that presented stimuli in a summary format (Buehner et al., 2003; Lu et al., 2008). Likewise, the experimental instruction may influence how subjects sample and evaluate the evidence. Matute (1996) found that subjects who were not provided information about an experimental hypothesis that there was no causal relationship provided higher causal strength judgments than those who were informed about this hypothesis.

Experimental factors also include the elicitation of assessments of casual beliefs. Commonly, the causal judgment is assessed by a single question after subjects observe the evidence. However, this approach assumes that causal belief is a mono-faceted construct, and can be perfectly elicited and expressed by a single numerical response. Recently, there has been increasing attention paid to the potentially different constructs of causal reasoning. Several constructs related to causal reasoning, such as causal induction goals, causal reasoning directions and causal valence have been distinguished. Discrepancies among model comparison results may arise if different experimental questions elicit assessments of different constructs. The purpose of this paper is to examine how responses vary across various causal judgment questions, and to test alternative explanations for the inconsistent results in model comparison studies. We will begin with reviewing the current theoretical explanations for the differences in the responses assessed by different questions.

### Causal Valences

A question with generative valence asks how likely the candidate cause increases the occurrence of an outcome. A question with preventive valence asks how likely the candidate cause decreases the occurrence of the outcome. Although the majority of studies in causal reasoning focused on reasoning with generative valence, the distinction between these two valences has been examined in a number of previous studies (Wu and Cheng, 1999; Dennis and Ahn, 2001; Buehner et al., 2003; Catena et al., 2004; Lu et al., 2008; Mandel and Vartanian, 2009; Baetu and Baker, 2012).

Several studies have shown that evidence confirming the generative valence may have stronger effects on subject’s causal reasoning than evidence confirming the preventive valence (Dennis and Ahn, 2001; Baetu and Baker, 2012). Catena et al. (2004) found that the frequency of the outcome was more positively associated with subjects’ perceived causal strength of a generative link than a preventive link. Finally, Mandel and Vartanian (2009) found that subjects who were presented with a hypothesis with generative valence overestimated the occurrence of the effect, which in turn increased the perceived causal strength. These studies suggest that reasoning for the two causal valences may have different underlying evidence processing mechanisms. People may be more influenced by change in the causal evidence when responding to a generative valence question than to a preventive valence question.

### Causal Induction Goals

The causal graphical model proposed by Waldmann et al. (1995) distinguishes two goals of causal reasoning. First, *causal structure induction* refers to the use of covariation information to decide if there is a causal link between the cause and the effect. The structure induction involves evaluating and comparing evidence for multiple causal structures against one another (Griffiths and Tenenbaum, 2005). A second goal is *causal parameter estimation*, which concerns the strength of the causal links. The aim is to answer the question, “What is the probability with which a cause produces an effect, in the absence of other causes?” (Cheng and Novick, 2005; Holyoak and Cheng, 2011).

The common measure of the causal structure judgment can be a probability judgment question, such as “How likely is it that a candidate cause did cause the effect” (e.g., Griffiths and Tenenbaum, 2005, Experiments 1 and 2). On the other hand, a strength judgment asks subjects to rate judge how strong the relationship is between the candidate cause and the effect (e.g., Buehner et al., 2003, Experiment 3; Hattori and Oaksford, 2007, Experiments 1 and 2; Lober and Shanks, 2000, Experiments 1–3; White, 2009). The two types of questions can yield distinct model fit results for various models (see Griffiths and Tenenbaum, 2005; Hattori and Oaksford, 2007).

Cheng and Novick (2005, p. 700) defined a causal strength question as “the probability that a candidate cause c produces e when c occurs.” Lu et al. (2008) implemented causal strength question in accordance with this definition. They found that a structure-focused model fitted the responses of a causal structure judgment better than a strength model, while a strength model fitted the responses of a strength judgment better than a structure model. However, this type of question also differed from the structure question in previous examples, in terms of the direction of reasoning, which is discussed in the next section.

### Reasoning Directions

A further two types of causal judgment questions can be distinguished in terms of the directions of reasoning. *Predictive reasoning* goes from the cause to the effect. A predictive judgment question asks “given the presence of the cause, how likely is it that an effect would occur?” *Diagnostic reasoning*, on the other hand, requires one to reason from the effect to the cause, asking “given the observed effect, how likely was the effect caused by the candidate cause?”

Some experimental questions used in previous studies asked for a predictive judgment, while others asked for a diagnostic judgment. Lu et al. (2008), Experiment 1 asked subjects to judge “How many of the genes will be turned on (the effect) when they are being exposed to the protein (the cause).” The question focused on predictive reasoning, as it asked for a prediction of the effect given the occurrence of the cause. On the other hand, the question in Anderson and Sheu (1995), Experiment 1 was ’How likely the drug [cause] used in that hospital is the cause of side effects [effect] in patients?’ This question focused on diagnostic reasoning, as it asked the subjects to judge the causal relationship given that the effect has occurred.

There is evidence that the two causal reasoning directions can be empirically differentiated (Tversky and Kahneman, 1980; Cummins, 1995; Fernbach et al., 2011). Fernbach et al. (2011) found a significant interaction between the judgment directions and the strength of the background causes that were not explicitly stated in the reasoning scenario. Subjects’ predictive judgments were higher than their diagnostic judgments when the background cause was stronger than the candidate cause. Conversely, their predictive judgments were lower than the diagnostic judgments when the background cause was weak. Cummins (1995) suggested that the consideration of alternative causes can promote doubt regarding the sufficiency of the candidate cause in producing the effect. Thus, the strong background cause could lower people’s diagnostic judgment of how likely the effect is due to the candidate cause. On the hand, Cummins (2014) suggested that subjects are more likely to retrieve disablers of the effect in predictive reasoning than in diagnostic reasoning. The lower ratings in predictive judgments when the alternative cause was weak could be due to the retrieval of the disablers in predictive reasoning.

An alternative explanation regards the inputs and assumptions implied by a diagnostic question versus a predictive question (Cheng and Novick, 2005). These inputs may involve the role of the background cause, and prior knowledge of the temporal precedence of the candidate cause. The assumption of the temporal precedence of the cause holds for a predictive question as it provides the presence of the cause in the question. This assumption may not hold for the diagnostic question. Thus a predictive reasoning can be greater than the diagnostic reasoning if the temporal precedence of the cause is not clear in the diagnostic question.

### The Current Studies

The accuracy of model prediction may depend on how well the causal judgment question matched the proposed construct, as variability in responses can rise when questions mixed that do not measure the same construct. This implies that the inconsistent results in model comparison studies may be due to artifacts in assessing causal beliefs. It has been noticed in previous studies is that subjects may have different interpretations for the same question (e.g., Cheng and Novick, 2005; Meder et al., 2014). Some current treatments for unifying subjects’ interpretations include using a *post hoc* question to filter out subjects, whose self-reported interpretation did not match the one that the researcher wanted (e.g., Meder et al., 2014). However, this practice would seem to oppose the fundamental goals in scientific psychological research on reasoning, namely to impartially observe, describe, and understand human reasoning in all its variability, fallibility, and complexity. Capturing the impacts of questions formats on the variability of the responses, may provide information on how some cues can systematically influence the question interpretation of people.

In the present paper, we accounted for the variability of the responses and examined how question formats may affect the responses of subjects. In Study 1, we investigated three questions used in previous studies. These included a structure induction question, a strength estimation question and predictive judgment question. All the three questions were assessed for each of the two causal valences. Study 2 extended the investigation based on the results in Study 1, and specifically assessed the effects of reasoning direction in the questions. In addition, we presented a model comparison study to examine how the question formats influence the fit of various models.

## Study 1

Study 1 was designed to assess the effects of judgment questions on subjects’ causal judgment. The experiment applied the experimental paradigm of Lu et al. (2008). Evidence of the causal relationship was presented to subjects in a summary format, to reduce the memory demand. We selected three experimental questions which may measure three different constructs: structure induction, strength estimation, and predictive judgment. We elicited both a point estimate of each causal judgment, and an interval estimate that assesses the degree of confidence of the subjects. We aimed to explore how responses can differ among the three types of questions.

### Method

#### Participants and Design

Seventy-three adults (36 males and 37 females) in the United States were recruited via a Qualtrics online survey panel, with a mean age of 43.32 (SD = 15.09). The study had two causal directions, three question types, and three levels of covariation information in a mixed design. Subjects were randomly assigned to the generative condition (*N* = 38) or the preventive condition (*N* = 35). The within-subject conditions included the levels of causal covariation and types of judgment questions.

#### Materials and Variable Manipulation

Subjects were instructed to pretend they were employees in a bio-genetic company. Their task was to evaluate the effects of certain types of proteins on the expression of genes. They observed the results of a series of fictitious experiments. The cover story stated that, for each of these experiments, DNA strands extracted from human hair were tested by the testing protein. Subjects were presented one set of DNA strands that had not been exposed to the testing protein, and one other set of DNA strands that had been exposed to the protein. Each set contained 16 DNA strands, such that the sample size was 32 in total across all conditions. The status of genes (on or off) was displayed differentiated by their colors. They answered the causal judgment questions regarding the relationship between the states of the genes (being on or off) and the presence or absence of the protein.

##### Covariance

We define *P*(*E*+|*C*-) as the probability of genes being on given the absence of the protein, while *P*(*E*+|*C*+) is the probability of genes being on given the presence of the protein. The *P*(*E*+|*C*+) and *P*(*E*+|*C*-) information is reversed in the preventive condition. Three covariation conditions in the generative condition included (a) C1:P(E +|C-) = 0.25,P(E +|C+) = 0.5; (b) C2:P(E +|C-) = 0.5,P(E +|C+) = 0.75; and (c) C3:P(E + |C-) = 0.25,P(E + |C+) = 0.75. **Table 1** displays the frequencies of the covariance events in each covariance condition. We chose the three conditions because the three covariance levels had different causal strength or judgment values predicted by a number of different causal models such as the contingency and causal power models. If the differences in the models in term of fitting the experimental data are associated with the artifact produced by differing question formats, we may observe a shift in variability of the casual ratings across different types of judgment questions.

**Table 1:** Covariance information in Experiment 1.

**Cause**	**Present**		**Absent**	
**Effect**	**Present**	**Absent**	**Present**	**Absent**
**Generative**
C1	8	8	12	4
C2	4	12	8	8
C3	4	12	12	4
**Preventive**
C1	12	4	8	8
C2	8	8	4	12
C3	12	4	4	12

##### Causal valence (preventive versus generative)

Subjects in the generative condition were shown a higher frequency of the effect in the presence of the cause (i.e., when genes were exposed to the protein) than subjects in the preventive condition. They were asked to evaluate the relationship between the protein and the turning on of the gene. Subjects in the preventive condition were asked to evaluate the relationship between the protein and the turning off of the gene.

##### Question types

The three causal judgment questions included a causal structure induction question, a strength estimation question, and a predictive judgment question. The structure question asked “How likely do you think the turning on of the gene is due to being exposed to the protein?” The structure question is also a diagnostic question, because subjects are asked to reason from the effect to the cause. The strength question asked “How strong is the relationship between the turning on of the gene and being exposed to the protein?” This question is commonly used in causal reasoning studies. Finally, the predictive question is as in Lu et al. (2008), “Suppose that there is a sample of 100 DNA strands. The gene is ON in all 100 strands. If all of them were exposed to Protein M, how many of them will be turned OFF?”

#### Procedure

Each subject completed three judgment blocks, with different covariation information in the evidence. The order of the blocks was randomized. In each judgment block, the presentation of the evidence was followed by the three judgment questions. Subjects answered the structure judgment first, then the strength judgment, and finally the predictive judgment.

For each judgment question, subjects provided a best estimate by rating on a scale of 0–100% for the structure question, and 0–100 for strength and predictive questions. They were also asked to estimate an interval around their best estimate by providing a minimum estimate and a maximum estimate, such that they were 90% confident that the interval contained the true probability/strength of the causal relationship. The interval width (IW) measures the level of confidence of subjects. This also allows for assessing the precision of the subjective probability distribution for the causal relationships.

#### Data Analysis

The ordinary linear models such as analysis of variance (ANOVA) assume that the dependent variable is normally distributed, which has unbounded support. The dependent variables in the majority of causal reasoning studies are double bounded numerical or probability ratings from, for example, 0 to 100 (or 0 to 100%). When the causal strength in the experimental stimuli approaches extreme values such as 0 or 100, the judgments provided by subjects can be highly skewed. Moreover, there is likely to be heteroscedasticity in the data, as change in the mean also can change the variance. Therefore, linear models such as ANOVA are inappropriate. Furthermore, ordinary linear models only focus on the mean of the dependent variable, and so are unable to capture any change in its dispersion. The dispersion is informative, as it can be an indicator on how much subjects agree in perceiving the causal relationships. Higher dispersion in the ratings suggests greater discrepancy among causal ratings, which might be an indicator of the discrepancies either in interpretations of the causal judgment questions, or in the ways that people reason about causal relationships.

In the current study, we apply generalized linear models (GLMs) using the beta distribution to overcome these issues and to provide more informative and robust analysis^1^. The beta GLM utilizes two submodels that can deal simultaneously with the predictors of location and the predictors of dispersion. A location submodel links the linear combination of predictors with the mean of the dependent variable via a logit link function. A positive coefficient in the location submodel indicates a positive relationship between the predictor and the mean of the dependent variable. A precision submodel links the predictors with the variance component of the dependent variable, and indicates how the variance of the dependent variable changes as function of the predictors. A positive coefficient in the precision submodel indicates greater precision, or smaller variance of the data. The precision of the responses variable can be a measure of the consensus among subjects. A greater precision indicates a greater homogeneity in the responses provided by subjects. For a more detailed introduction to beta regression, readers may consult Smithson and Verkuilen (2006).

We applied beta GLMs to model the best estimate, as well as the interval estimates. In each model, effects coding was applied to create dummy variables for each factor (see Table notes for each model estimation results) such that the intercepts of the two submodels represent the overall mean and overall precision of the causal ratings. Effects coding also resolved the potential problem of muticollinearity in the interaction and their constituent main-effects terms (Tabachnick and Fidell, 2007).

We used Bayesian Markov Chain Monte Carlo methods for the model estimation and inference, using OpenBUGS (version 3.2.2). In all following analysis, we drew 8000 samples from the posterior distributions, with an initial burn-in of 4000 iterations. We used Deviance Information Criteria (DIC, Spiegelhalter et al., 2002) for model comparison and selection. DIC is a combination of a measure of model fit (the mean difference between the log-likelihood of the tested model and a saturated model) and a penalty measure of model complexity. A smaller DIC indicates a better model. In general, a difference of 3–7 units in DIC indicates the evidence of a substantial difference in model fit of the two models, while a change of over 10 units is a definite indication that the model with smaller DIC is better than the model with the higher DIC (Spiegelhalter et al., 2002). We then report the estimation results for the best model, including the estimated means, standard errors and 95% credibility interval (CI) of the coefficients of the two submodels. The 95% CI includes the 95% interval of the posterior distribution of the parameter, and can be interpreted in a similar sense as a 95% confidence interval in traditional statistical analysis.

### Results

**FIGURE 1:**
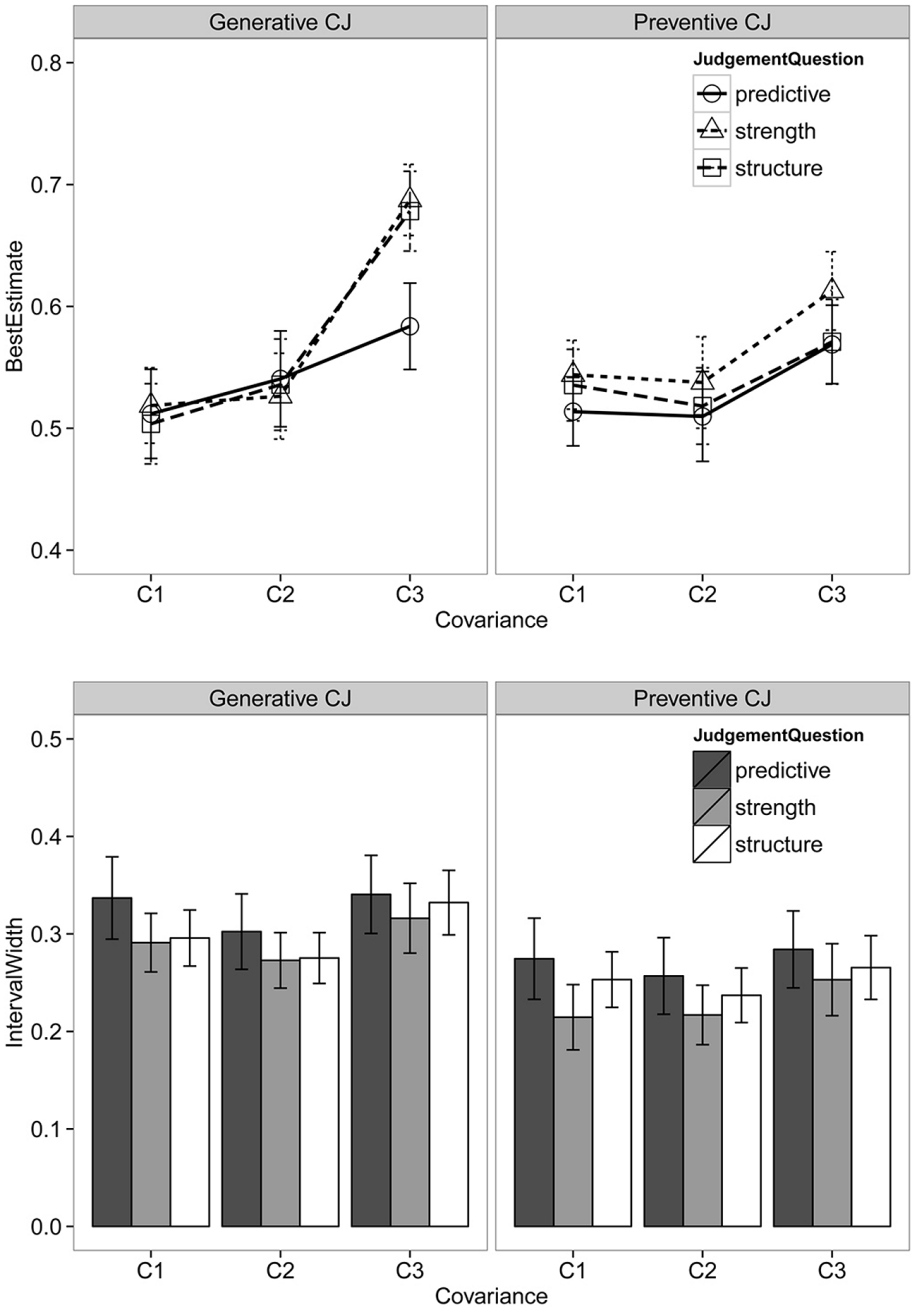
**Best estimates (**upper**) and interval widths (**lower**) for different judgment questions across the two causal valence conditions**. Error bars are standard errors.

There were 657 responses for each type of estimate (best, minimum, and maximum). Some subjects had the invalid minimum and maximum estimates (i.e., provided a minimum estimate greater than the maximum) for one or two questions. These invalid responses were removed from the analysis, leaving 635 valid responses. **Figure 1** summarizes the ratings of best estimates and the IW of subjects in the two causal valence conditions.

#### Best Estimate

**Table 2** summarizes the contribution of each factor to the model by comparing the DIC value of a model with that factor and a model without that factor. Covariance had most substantial effects on subject’s causal ratings, both on the mean (ΔDIC = 61.4) and precision of the causal ratings (ΔDIC = 17.7). Removing causal valence from the precision submodel decreases the model fit by 26.1 DIC units, suggesting causal valence had a substantial main effect on the precision of the judgment distribution. Finally, the ΔDIC values of 18.5 and 15 suggest that question*s* types had substantial main effects on both the mean and the precision of the rating distributions. To evaluate the interaction effects, a model with an interaction added was compared with a model without that interaction. Only the interaction between causal valence and covariance had a substantial contribution to the main effect model.

**Table 2:** ΔDIC of effects of causal valence, question types, and covariance on best estimates in Study 1.

Removed factor		
Location submodel	Precision submodel	Δ DIC
Causal valence		-1.4
Question type		18.5
Covariance		61.4
	Causal valence	26.1
	Question type	15
	Covariance	17.7
Causal valence × Covariance		8.3

*ΔDIC compares a full model with a model without a factor, and indicates the contribution of the removed factor. A positive ΔDIC suggests that removing the factor decreases the model fit. The Main-effect model included all three factors (causal valence, question type, and covariance) in both the location submodel and the precision submodel. No other interactions were found to be significant; those interactions not included in the table*.

The results of parameter estimation for the final model are summarized in **Table 3**. In the following analysis, we report the results of the three factors – covariance, causal valence, and question types in that order. For each factor, we will first report the results of the location submodel, and then the results of the precision submodel. We then report the interaction effects between different factors.

**Table 3:** Random effect beta GLM of best estimates predicted by causal valence, question types, and covariance in Study 1.

Variables	Parameter	Coefficient	SE	2.5%	97.5%
Random intercept		0.57	0.10	0.38	0.76
**Location submodel**
Intercept	*b*_0_	0.26	0.07	0.12	0.40
Causal valence	*b*_1_	-0.06	0.07	-0.19	0.10
Covar2	*b*_2_	-0.13	0.04	-0.20	-0.05
Covar3	*b*_3_	0.27	0.04	0.20	0.34
Structure	*b*_4_	0.00	0.04	-0.08	0.07
Predictive	*b*_5_	-0.06	0.04	-0.14	0.01
Covar2 × Causal valence	*b*_6_	0.00	0.04	-0.07	0.08
Covar3 × Causal valence	*b*_7_	-0.10	0.04	-0.17	-0.02
**Precision submodel**
Intercept	*d*_0_	2.20	0.06	2.08	2.30
Causal valence	*d*_1_	0.29	0.06	0.18	0.40
Covar2	*d*_2_	-0.17	0.09	-0.34	0.02
Covar3	*d*_3_	0.07	0.09	-0.10	0.24
Structure	*d*_4_	-0.01	0.08	-0.18	0.15
Predictive	*d*_5_	-0.18	0.09	-0.36	0.01

*Causal valence is coded as -1 = generative condition and 1 = preventive condition. The two dummy variables for covariation conditions: Covar2 is coded as -1 = C1, 1 = C2, and 0 = C3 condition. Covar3 is coded as -1 = C1, 0 = C2, and 1 = C3 condition. The coefficients of Covar2/Covar3 represent the difference between the ratings in the C2/C3 condition and the overall mean/precision of the causal ratings. The two dummy variables for question type conditions: Structure is coded as -1 = strength judgment, 1 = structure judgment, and 0 = predictive judgment; predictive is coded as -1 = strength judgment, 0 = structure judgment, and 1 = predictive judgment. The coefficients of Structure/Predictive represent the difference between the ratings in the Structure/Predictive question and the overall mean/precision of the causal ratings. 2.5 and 97.5% are the lower and upper bounds of the 95% credibility interval*.

The causal estimates of the C2 condition were significantly lower than the grand mean, while the ratings of C3 condition were significantly higher than the grand mean (see **Table 2**). A *post hoc* repeated comparison^2^ suggested that the mean of C2 estimates did not significantly differ from C1 (*b* = 0.01, 95% CI = [-0.11, 0.14]), whereas the mean of the C3 condition was significantly higher than both C1 (*b* = 0.32, 95% CI = [0.23, 0.40], odds ratio = 1.38) and C2 (*b* = 0.41, 95% CI = [0.24, 0.41], odds ratio = 1.51) condition. The odds ratio at 1.38 indicates that subjects perceived the causal relationship between the protein and the status of genes in C3 condition as having 1.38 times greater odds than in the C1 and C2 conditions). Regarding the effects of covariance on the precision of rating distribution, both C2 and C3 did not have significant different precision from the overall grand mean precision. The *post hoc* comparison suggested that the effect of covariance on the precision was mainly contributed to by the difference between C2 and C1 conditions, where the responses of the C1 condition was significantly more homogeneous than the C2 condition (*d* = 0.32, 95% CI = [0.07, 0.56]).

There was no significant difference in the mean ratings between the two causal valence conditions. However, the rating distribution of the preventive condition was significantly more precise than the generative condition, suggesting that subjects in the preventive condition provided more homogeneous ratings than those in the generative condition (*d*_1_ = 0.29, 95% CI = [0.18, 0.40], see **Table 2**). In addition, there was a significant interaction between covariance and causal valence, where the difference between C3 and C1 was greater in the generative condition than in the preventive condition.

Next, the *post hoc* comparison revealed that the effect of question type on the mean of the ratings was mainly contributed to by the higher and more precise ratings of the strength question compared with the predictive question (*b*= 0.12, 95% CI = [0.02, 0.25], odds ratio = 1.14; *d* = 0.35, 95% CI = [0.02, 0.66]). The structure induction question did not significantly differ from either the strength question (*b* = 0.04, 95% CI = [-0.09, 0.20]; *d* = 0.16, 95% CI = [-0.10, 0.40]), or the predictive question (*b* = 0.06, 95% CI = [-0.07, 0.18]; *d* = 0.16, 95% CI = [-0.15, 0.47]).

#### Confidence Interval Widths

**Table 4** summarizes the contribution of each variable to explaining the variation in the IWs, and **Table 5** displays the estimation results the final model. Covariance had substantial contribution to the location submodel. The *post hoc* comparison suggested that the effect of covariance was contributed to by the difference between C2 and C3, where the mean IWs of C2 condition was significantly wider than C3 condition (*b*_2_ = 0.12, 95% CI = [0.04, 0.19], odds ratio = 1.13). The IWs of C1 were not significantly different from either C2 or C3 condition. Covariance also had substantial contribution to the precision submodel, and the effect of covariance was moderated by causal valence.

**Table 4:** ΔDIC of effects of causal valence, question types, and covariance on interval estimates in Study 1.

Removed factor		
Location submodel	Precision submodel	Δ DIC
Causal valence		0.0
Questions		49.7
Covariance		25.5
	Causal valence	4.0
	Questions	61.4
	Covariance	14.4
	Causal valence × Covariance	7

*The main-effect model included all three factors (causal valence, question type, and covariance) in both the location submodel and the precision submodel*.

**Table 5:** Random effect beta GLM of interval estimates predicted by causal valence, question types, and covariance in Study 1.

Variables	Parameter	Coefficient	SE	2.5%	97.5%
Random intercept		0.57	0.10	0.38	0.76
**Location submodel**
Intercept	*b*_0_	-1.06	0.13	-1.28	-0.80
Causal valence	*b*_1_	-0.21	0.10	-0.40	0.00
Covar2	*b*_2_	0.12	0.04	0.04	0.19
Covar3	*b*_3_	-0.11	0.04	-0.18	-0.03
Structure	*b*_4_	-0.10	0.04	-0.18	-0.02
Predictive	*b*_5_	0.28	0.05	0.19	0.38
**Precision submodel**
Intercept	*d*_0_	2.13	0.06	2.02	2.24
Causal valence	*d*_1_	0.04	0.08	-0.12	0.19
Covar2	*d*_2_	0.05	0.09	-0.11	0.22
Covar3	*d*_3_	-0.08	0.08	-0.24	0.07
Structure	*d*_4_	0.55	0.09	0.38	0.73
Predictive	*d*_5_	-0.74	0.10	-0.92	-0.54
Covar2 × Causal valence	*d*_6_	-0.15	0.06	-0.27	-0.03
Covar3 × Causal valence	*d*_7_	0.18	0.08	0.03	0.33

*Causal valence is coded as -1 = generative condition and 1 = preventive condition. The two dummy variables for covariation conditions: Covar2 is coded as -1 = C1, 1 = C2, and 0 = C3 condition. Covar3 is coded as -1 = C1, 0 = C2, and 1 = C3 condition. The coefficients of Covar2/Covar3 represent the difference between the ratings in the C2/C3 condition and the overall mean/precision of the causal ratings. The two dummy variables for question type conditions: Structure is coded as -1 = strength judgment, 1 = structure judgment, and 0 = predictive judgment; Predictive is coded as -1 = strength judgment, 0 = structure judgment, and 1 = predictive judgment. The coefficients of structure/predictive represent the difference between the ratings in the structure/predictive question and the overall mean/precision of the causal ratings*.

The IWs did not significantly differ between the two causal valences. Causal judgment questions, on the other hand, made significant contributions to both submodels. The *post hoc* repeated comparison revealed that strength judgment did not significantly differ from structure judgment in terms of the mean IWs, however, strength judgment had significantly less homogeneity in IWs than structure judgment (*d*= -0.40, 95% CI = [-0.72, -0.10]). Predictive judgment had significantly larger and more heterogeneous intervals than both the structure judgment (*b* = 0.38, 95% CI = [0.24, 0.52]; *d* = -1.28, 95% CI = [-1.61, 0.97]) and the strength judgment (*b* = 0.47, 95% CI = [0.30, 0.61]; *d* = -0.91, 95% CI = [-1.23, -0.60]).

### Discussion

The results showed that question formats had significant effects on both the mean and variability of the best estimates provided by subjects. The effects were observed in both causal valences and all three levels of covariance. It was also found that the difference between the predictive question and the other two questions (i.e., strength question and structure induction question), were larger than the difference between the other two questions.

One explanation is that the predictive question motivated predictive reasoning, while both the structure question and strength question focus subjects’ attention on the current evidence, which may motivate diagnostic reasoning. The difference might be due to subjects underestimating the effects of alternative causes and being more likely to retrieve disablers (Cummins, 2014). However, the number of alternative causes and disablers people can retrieve is positively associated with their familiarity with the reasoning context (Cummins, 1995). This implies that novel and abstract scenarios, which may severely limit the number of alternative causes that can be imagined, should result in similar ratings between diagnostic judgments and predictive reasoning.

An alternative explanation can be related to the differences in the grammatical tense. Both the structure and the strength question used the present tense and might direct subjects’ attention to the evidence that was presented to them. The predictive question, on the other hand, was in future tense and may encourage subjects to judge to what extent the causal relationships in the current observations can be generalized to future instances. The distances between the current observations and the future instances contribute to the difference between the predictive question and the other two questions. Further discussion of the effect of reasoning directions will be presented in Study 2A. In addition, subjects in the present study responded to the predictive question after the structure question. The fixed order of the two questions might influence subjects’ responses. The problem of order effects was also addressed in Study 2A by endorsing a between-subject design to test the differences between these two questions.

Furthermore, we also found question formats had significant effects on the mean and variability of the in IWs. The predictive question had significantly wider IWs than the other two questions, suggesting that subjects had lower confidence in predictive judgment than they did in the other two judgments. All three questions had significant difference in the variability of the IWs, suggesting that question formats can influence the degree of individual differences in confidence interval estimates. One possible explanation for this finding relates to the difference in the response modes required in the three questions. The structure induction question requires a probability judgment, the strength judgment requires a numerical judgment, and the predictive question required subjects to make a frequency judgment. Extensive evidence from judgment and decision making literature shows that the level of self-reported confidence resulting from interval elicitation can be sensitive to the response modes (Juslin et al., 1999; Moore and Healy, 2008; Fellner and Krügel, 2012). People are more resistant to overconfidence when making frequency judgments than probability judgments as fewer computational steps may be required in the natural frequency estimates (Gigerenzer and Hoffrage, 1995). Thus, the wider interval estimates of the predictive judgment, which suggests a lower confidence level, might be an artifact of lower overconfidence in frequency judgments.

Finally, we found the increase in the causal strength of the evidence had greater positive influence on the estimates of the subjects in the generative condition than those in the preventive condition. This finding is in line with previous studies, suggesting that causal valence can influence the evidence processing. A more interesting finding is the greater homogeneity of the ratings in the preventive condition than the generative condition. According to Cheng’s (1997) power model, humans have the prior assumption that the background cause is always generating the effect. The background cause and the candidate cause have the same causal influence directions if the candidate cause is a generative cause; while they are in opposite directions if the candidate cause is a preventive cause.

The significantly higher variability of the ratings in the generative condition may suggest an interaction between the perceived causal strength of the background cause and the estimated causal strength of the candidate cause. In the generative condition, the background cause and the candidate cause are competing for the generative influence on the effect events. Subjects’ causal estimates are influenced by their perceptions of both the link between the candidate cause and effect, and the link between the background cause(s) and the effect. On the other hand, in the preventive condition subjects’ estimates are only influenced by perceptions of the link between the candidate cause and effect, as the background cause does not compete for the preventive causal strength. Estimates that have two influences are likely to vary more than estimates that have just one influence, and thus may account for the greater variability of the strength estimates in the generative condition.

## Study 2A

Study 1 revealed that question formats can have substantial impacts on the response distributions of subjects. We do not have a clear explanation for the difference between the predictive judgment question and the other two questions. One explanation for the difference between the predictive question and the other two questions regards the impact of reasoning directions elicited by different questions. The structure induction question may elicit diagnostic reasoning by subjects, while the strength question does not impose any particular reasoning direction. The distinction between diagnostic and predictive reasoning has not been directly assessed in Study 1 due to two confounding factors including the grammatical tenses and the response mode. In addition, subjects’ responses to the predictive question might be influenced by the other two questions due to the order of the question presentations Study 2A was designed to explore the impacts of diagnostic versus predictive judgments elicited by question formats by isolating the factor of judgment directions. The question type was treated as a between-subject condition to minimize the interference between different types of questions. Subjects were presented either a diagnostic judgment question or a predictive judgment question. The questions in both conditions had the same grammatical tense and response format. If the difference between the predictive question and the other two questions was due to the judgment directions in the question, we expected to observe a significant difference between two judgment direction conditions.

### Method

A total of 100 subjects were recruited via an online crowd-sourcing service CrowdFlower. Subjects were paid 50 cents (USD) for their participation. The study had a 2 causal valences × 2 judgment directions between-subject design. The setting of the experiment was the same as in Study 1. Subjects observed the status of a particular protein and the expression of the gene, and then made the judgments about the effects of the protein on the expression of the gene.

Subjects in the predictive condition were asked “Suppose a randomly selected sample of DNA strands with the particular gene being on is exposed to this protein, how likely do you think this particular gene in that sample would be turned off?” Subjects in diagnostic judgment conditions were asked “Suppose a random selected sample of DNA strands has the particular gene being off after being exposed to this protein. How likely do you think the turning off of the gene will be due to exposure to this protein?” It was noticed in Study 1 that the two causal valences differed in their mean responses for the C1 and C2 condition, but not for the C3 condition^3^. Because the covariance levels and causal valences were not the main focus of the study, only the C3 covariance condition was selected.

### Results and Discussion

**FIGURE 2:**
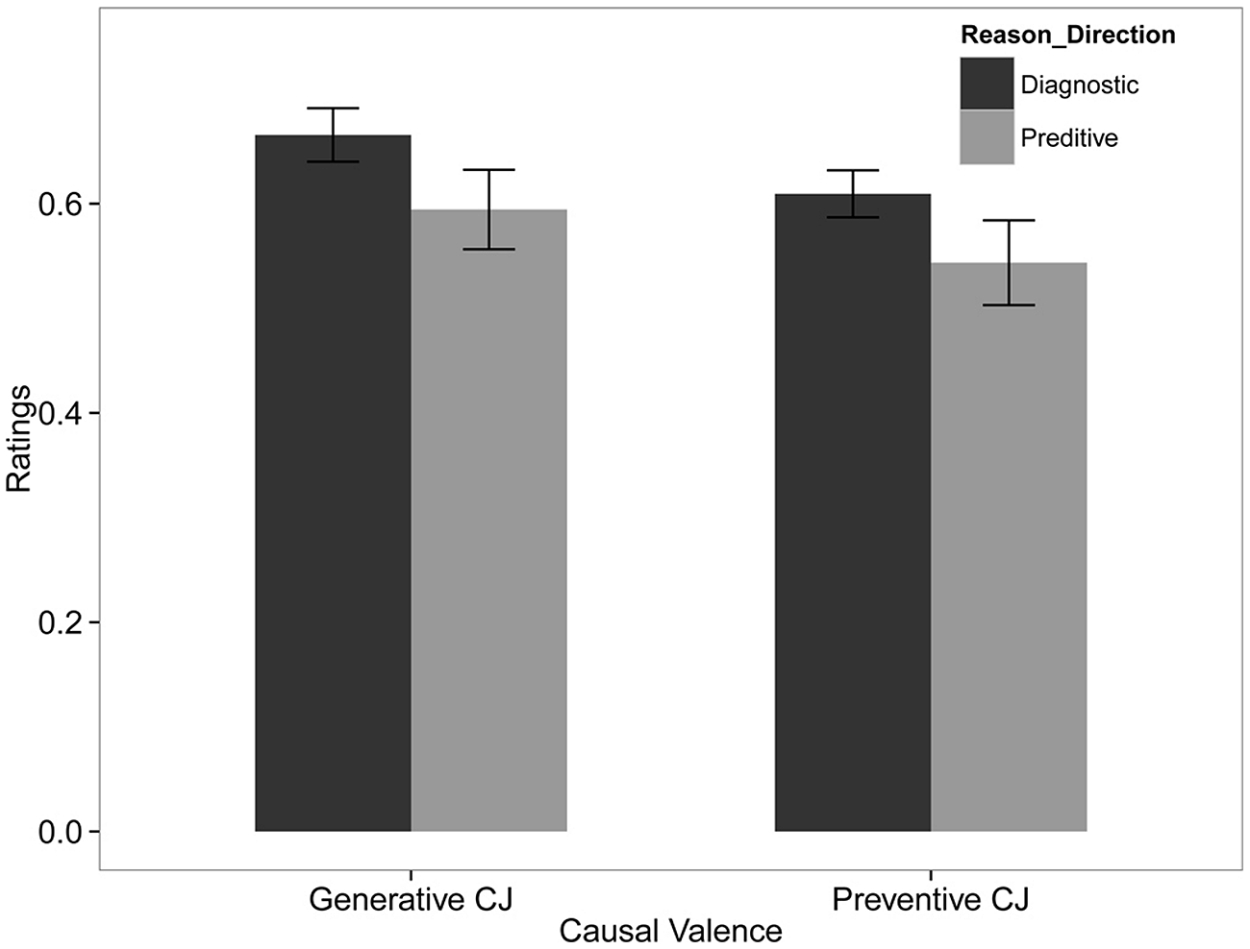
**Best estimates for the two causal valence and reasoning direction conditions. Error bars are standard errors**.

**Figure 2** summarizes the mean and standard error of subjects’ estimates. **Table 6** and **Table 7** summarize the result of the beta GLM for the mean ratings of causal relationship. The responses of subjects who were asked a question with diagnostic reasoning direction were significantly higher and more precise than those who were asked a question with predictive reasoning direction (*b*_1_ = 0.16, 95% CI = [0.02, 0.29], odds ratio = 1.17; *d*_1_ = 0.36, 95% CI = [0.09, 0.69]). Causal valence had no significant effects on the mean or precision of subjects’ responses.

**Table 6:** ΔDIC of effects of reasoning directions and causal valences on best estimates in Study 2.

Removed factor		
Location submodel	Precision submodel	Δ DIC
Causal valence		1.4
Reason direction		4.3
	Causal valence	-2.02
	Reason direction	5.38
Causal valence × Reason direction	Causal valence × Reason direction	-4

**Table 7:** Beta GLM of best estimates predicted by reasoning directions and causal valences in Study 2.

Variables	Parameter	Coefficient	SE	2.5%	97.5%
**Location submodel**
Intercept	*b*_0_	0.40	0.07	0.27	0.54
Reasoning direction	*b*_1_	0.16	0.07	0.02	0.29
Causal valence	*b*_2_	-0.1	0.07	-0.24	0.03
**Precision submodel**
Intercept	*d*_0_	2.16	0.14	1.89	2.42
Reasoning direction	*d*_1_	0.36	0.14	0.09	0.63
Causal valence	*d*_2_	0.01	0.13	-0.25	0.28

*Causal valence is coded as -1 = generative condition and 1 = preventive condition. Reasoning direction is coded as -1 = predictive reasoning and 1 = structure reasoning*.

The results in Study 2 provide evidence for the difference between the two reasoning directions by controlling the tenses and belief elicitation methods. Results replicated the finding in Study 1, where the diagnostic judgments were higher than the predictive judgments. Unlike the realistic and familiar scenarios used in previous studies, the current study applied novel and abstract scenarios, which may severely limit the number of alternative causes that can be imagined. While this explanation may account for equality in diagnostic and predictive ratings, it does not explain our finding that the mean in the diagnostic condition was higher than the mean in the predictive condition.

Previous studies applied realistic reasoning scenarios that provide prior knowledge of the temporal precedence of the candidate cause and possible alternative causes, which in turn facilitate diagnostic reasoning. Prior knowledge of the temporal precedence of the cause strengthens links between the cause and the effect (Cobos et al., 2002). For example, one of the statements in Fernbach et al. (2011) Experiment 1 asked “A newborn baby is drug addicted. How likely is it that its mother is drug addicted?” Belief in the temporal precedence of “mother is drug addicted” over “a newborn baby is drug addicted” may contribute to the strength of the judged causal link. Thus, when there is no explicit statement in diagnostic reasoning, subjects may vary their assumption about the precedence of the cause depending on the scenario. This question was addressed in Study 2B.

## Study 2B

Study 2B aimed to investigate how an explicit cue regarding the temporal precedence of the cause (TPC) could influence the diagnostic judgment. In addition, it examined the variability in assumptions about TPC among subjects who were presented a question without the explicit cue, and how those assumptions could affect the final responses.

### Method

A total of 157 subjects (106 females; mean age at 38.54, SD = 12.57) were recruited via the online crowd-sourcing platform CrowdFlower, and were paid 10 cents (USD) for participation. Forty subjects completed the diagnostic question with a TPC cue (identical to the Study 1A diagnostic condition). Responses of those subjects and the 57 subjects in the diagnostic condition in Study 2A were combined and served as the condition with the TPC cue (48 in generative condition and 46 in preventive condition).

The remaining 117 subjects (56 in the generative condition and 61 in the preventive condition) were assigned into a condition without the TPC cue. The experimental setting was the same as Study 2A. Subjects were shown the covariance information at the C3 contingency level. The subsequent judgment question was a diagnostic question without the explicit cue of the temporal precedence of the cause: “Suppose there is a new randomly selected sample DNA, with the particular gene on (*off* for preventive condition). How likely do you think the turning on (*off* for preventive condition) of this particular gene in that sample DNA is due to the exposure to this chemical?” On a separate page, subjects in the no TPC cue condition were asked to rate how likely that the new sample DNA has been exposed to the testing chemical.

### Results

A beta GLM was first conducted to examine the impact of the presence versus absence of the TCP cues on causal reasoning. Results suggest that subjects who were provided the TCP cue had generally higher ratings than those who were not provided the TCP cue (*b*_1_ = 0.21, 95% CI = [0.00, 0.46]). Also, subjects who were provided the TCP cue provided significantly more homogenous responses than those were not provided the TCP cue (*d*_1_ = 0.69, 95% CI = [0.34, 1.04]). Causal valence had no significant effects on the mean or precision of subjects’ responses. There were also no significant interactions between causal valence and the presence of the TCP cue in either submodel.

We conduct a correlation analysis for the responses to the diagnostic question without the TCP cue and the degree of the belief in the temporal precedence of the cause. There was a significant positive correlation between the causal judgments and the degree of the belief in the temporal precedence of the cause (*r* = 0.48, *p* < 0.001), for both the generative condition (*r* = 0.51, *p* < 0.001) and preventive condition (*r* = 0.44, *p* < 0.001). Beta regression analysis showed that the increase in the belief of the precedence of the cause significantly contribute to the higher mean and precision of causal judgments (*b*= 2.25, 95% CI = [1.56, 3.00], odds ratio = 9.49; *d*= 2.52, 95% CI = [1.47, 3.56]). There was no significant interaction between the belief in the cause precedence and causal valence.

### Discussion

The results demonstrated that subjects varied in their presumption of the temporal precedence of the cause when they were not provided the explicit information about the precedence. The extent to which they believed in the precedence correlated positively with their beliefs in the causal relationship. In Studies 1 and 2A, the explicit cue of the cause precedence in the diagnostic question, in addition to the limit in the number of alternative causes, could contribute to the higher ratings on a diagnostic question than a predictive reasoning question. This finding also provides an alternative explanation for the conflicting experimental findings in Fernbach et al. (2011). The results also suggested that the precedence of the cause may not be a default assumption held by subjects, and the reasoning scenario may influence the degree to which subjects adopt this assumption. The absence of a TCP cue may inflate the variation in causal ratings among subjects.

## Impact of Question Formats on Model Fit

To demonstrate the effects of question formats on assessing model fit, we fitted several computational models to the data in Experiment 1. The models selected for the current study include the causal power model (Cheng, 1997; Lu et al., 2008), the causal attribution model (Cheng and Novick, 2005; Holyoak et al., 2010), and the structure induction model for both diagnostic reasoning and predictive reasoning (Meder et al., 2014). These models have been detailed in Meder et al. (2014). We chose these models because they were the only models that may distinguish different types of causal queries (see the model introductions that follow). We aimed to examine the extent to which a model maps its proposed construct. We emphasized how model fits varied across the different questions rather than discussing which model is the best model.

### MLE versus Bayesian Extension

Early normative models of causal reasoning from covariation information included ΔP (Jenkins and Ward, 1965) and power PC model (Cheng, 1997). Both ΔP and power PC maximize the likelihood of the observed data by regarding the model probabilities as equivalent to the empirical probabilities (Griffiths and Tenenbaum, 2005). Both models were criticized for ignoring model uncertainty and the prior beliefs of the reasoner (Griffiths and Tenenbaum, 2005; Meder et al., 2014). Griffiths and Tenenbaum (2005) applied a Bayesian framework and introduced the causal support model to account for model uncertainty in causal reasoning. The causal support model compares the posterior probability of a candidate causal structure against the posterior probability of a null causal structure (see Griffiths and Tenenbaum, 2005 for details). The initial Bayesian implementation uses a uniform prior probability distribution over the causal structures and over the parameters. Lu et al. (2008) extended the Bayesian framework by introducing a Strong-and-Sparse (SS) prior distribution for both the parameters and the causal structures. The rationale is that human have strong preferences for the simple causal structure and maximizing the strength of each individual cause. In the current model fitting study, we tested the following causal models with each of the three estimation methods: maximum likelihood estimate (MLE), Bayesian estimation with uniform prior distributions, and Bayesian estimation with SSprior distributions.

### Conditional Probability and Structure Induction Models

One way to model the diagnostic and predictive reasoning is to use the conditional probabilities. The diagnostic probability *P*(C| E) is the probability of observing the cause given that the effect has been observed, while the predictive probability *P*(E| C) is the probability of observing the effect given that the cause has been observed. Meder et al. (2014) introduced a Bayesian extension, namely the Structure Induction Model, to account for model uncertainty and the prior beliefs of the reasoner. The Structure Induction Model for a diagnostic judgment averages the posterior probability of a diagnostic probability given the posterior probability of the candidate causal structure, and the posterior probability of a diagnostic probability given the posterior probability of the alternative causal structure. Similarly, the Structure Induction Model for a predictive judgment averages the posterior probabilities for the predictive probability.

### Causal Attribution

Cheng and Novick (2005) proposed the concept of “causal attribution” in contrast to “causal power.” A measure of causal attribution differs from a measure of causal power in terms of the input and assumption provided in the reasoning context. One such difference between two concepts regards whether the occurrence of the cause is observable. As we have explored in Experiment 3, structure diagnostic reasoning can be sensitive to the assumption of the observable status of the cause. Cheng and Novick (2005) discussed several queries relating to causal attribution. The one that may be closest to the structure question in Experiment 1 is the causal attribution to the candidate cause given that both the cause and effect have been observed, thus the model corresponding to that query is tested in the current study (see Eqs 6–7 in Cheng and Novick, 2005, p. 701).

### Method, Results, and Discussion

Some previous model comparison studies (e.g., Lu et al., 2008) evaluated models by correlates the model predictions with the mean of human’s judgments. This approach directly ignores the impacts of the variability of the ratings. In the current study, we applied the mean square errors (MSEs) to capture the deviation between the models’ predictions and each of the subjects’ ratings. In addition, MSEs were not calculated for the causal attribution model and structure induction model to the causal ratings in the preventive valence condition, because both of the models currently only accounted for the generative causal reasoning. Chi-square statistics were calculated to test whether the MSEs were significantly different between different question formats. We compared the MSEs both across all models, and within each model. A significant result of the chi-square test suggests the fit of model(s) was significantly different between different questions.

The MSE and bootstrapping results of MSE for each model variation are available in the online supplemental materials. First, all models fitted the strength question best, while fitting the predictive question worst. MSEs of the predictive judgments were significantly higher than the structure judgments (χ^2^ = 19.69, *p* < 0.001)^4^, and the strength question (χ^2^ = 30.53, *p* < 0.001). MSEs for the structure question were also higher than the strength question (χ^2^ = 10.84, *p* = 0.001). There was no evidence that the causal power model and structure induction model for predictive reasoning fitted the predictive judgments better than the diagnostic reasoning model. The patterns of the MSEs across the three types of questions were similar among different types of models and their variants. These results, in fact, reflect the impact of variability of the ratings on model fit. The predictive judgments had highest variability among all three types of questions, while the variability of the ratings in the generative condition was higher than the preventive condition. As none of the models account for the variability of the ratings, the question formats, which had a significant impact on the rating variability, in turns, had significant impact on the model fits.

## General Discussion

The three studies demonstrated that subjects’ responses to a question about causality are influenced by how the question is phrased. Study 1 examined three different questions used in previous causal judgment studies. The question formats had substantial effects on the mean and variability of both the best estimates and interval estimates. Study 2A demonstrated the impacts of the reasoning directions in the questions on causal judgments. A question with a diagnostic reasoning direction produced significantly higher mean responses than a question with a predictive reasoning direction. Study 2B revealed that subjects’ prior belief in the precedence of the cause, which can be influenced by a cue in the question, can significantly influence their ratings on a diagnostic question.

While the results in Study 2B could explain the higher ratings in the diagnostic judgments than the predictive judgments, the higher variability in predictive judgments than the diagnostic judgments remains unexplained. One explanation is that there were greater individual differences in confidence on evaluating an event that has not occurred. Alternatively, it has been argued that people may interpret a diagnostic query in different ways depending on how they attribute the effects to the candidate model in relation to the alternative or background cause (Cheng and Novick, 2005; Meder et al., 2014). The higher variability in predictive judgments implies that people may also interpret a predictive question in different ways. For example, the predictive question might be interpreted as “how likely the gene would be turned on [by the chemical alone],” alternatively, it can also be interpreted as “how likely the gene would be turned on [by both the chemical or the unobservable cause].” The first interpretation can result in an evaluation of *P*(E| C), while the later one can results in an evaluation of *P*(E| C, B). Nevertheless, further research is required to understand the factors associated with the individual differences in predictive judgments.

These variations in causal estimates revealed by the three studies can partially explain the inconsistent model comparison results in the previous causal judgment studies. In our final model fitting study, the fitting statistics for different models were significantly influenced by the question types. Most models, regardless their proposed constructs, fitted a strength question best while fitted the predictive question worst. This may reflect the fact that the predictive judgments had much larger variability than the other two judgments.

## Conclusion

The present paper explored how responses in causal reasoning can be influenced by the types of questions. If a model yields good fit for responses to a particular question type, it may imply that the model may map onto a particular aspect of the construct measured by that question. However, we suggest that future research in causal reasoning and judgment should place a high priority on disentangling the effects of question formats from the effects of experimental manipulations, because that will enable comparisons between models of causal reasoning uncontaminated by method artifact.

The impacts of question formats have been largely neglected in previous studies. One major reason is that the previous studies paid little attention to the fact that people are not perfect in numeric and probability judgments when expressing their beliefs. Therefore the question response modes can contribute to apparently inconsistent causal ratings from human subjects. None of the previous studies has paid attention to the variability of the causal ratings, perhaps as a consequence of not applying appropriate statistical analysis methods. Ignoring the violation of statistical assumptions such as homogeneity of variance and normality risks, misleads researchers’ interpretation of the results, as well as neglecting important information in the data. The current studies addressed these issues, and the beta GLM provided more appropriate statistical analyses.

We discussed our results from the perspective of the differences in causal constructs and assumptions. Future studies may examine the impacts of other questions format factors. For example, a frequency judgment, a probability judgment and a likelihood odds judgment may differently modulate between-question variability. People may be less familiar with odds than with relative frequencies, thus using questions that assess the odds judgments may increase response variability and decrease response validity (Price, 1998). Furthermore, applying 95% confidence intervals as a measure of confidence level might be limited by the ability of the reasoners to understand and elicit the upper and lower bound of the confidence interval. It has been suggested that people may provide may not have sufficient adjustment and provide narrow interval estimates (Juslin et al., 1999; Soll and Klayman, 2004). The variability in interval estimates may be resulted by individual difference in adjustment, which depends on the formats of elicitation and the judgment domain. Future studies may use alternative confidence measures such as adaptive interval adjustment procedure or certainty equivalence to batter capture the confidence levels. For example, the adaptive interval adjustment procedure proposes a specific interval around subjects’ best estimates and asks subject how they are confident that the true probability falls into the given interval (Winman et al., 2004).

## Conflict of Interest Statement

The authors declare that the research was conducted in the absence of any commercial or financial relationships that could be construed as a potential conflict of interest.

## Footnotes

1 A more detailed introduction of our modeling approach is provided in the online supplemental material at: http://goo.gl/E2dckt.

2 The *post hoc* comparison applies dummy coding that allows for the direct comparisons between two individual factor levels. For example, to test the difference between C1 and C2 conditions, the ratings in C1 was coded as 0 for the dummy variable C2. The coefficient of the dummy variable C2 indicates the difference between the C1 condition and C2 condition.

3 The pattern of difference matched the pattern predicted by the causal power model.

4 The χ^2^ statistics was calculated by taking the difference between the summed MSEs of one comparison level versus the other level.
